# Protective effects of ethyl pyruvate on sperm quality in cyclophosphamide treated mice

**Published:** 2015-05

**Authors:** Zahra Bakhtiary, Rasoul Shahrooz, Abbas Ahmadi, Leila Zarei

**Affiliations:** 1 *Department of Comparative Histology and Embryology, Faculty of Veterinary Medicine, Urmia University, Urmia, Iran.*; 2 *Solid Tumor Research Center, Urmia University, Urmia, Iran.*

**Keywords:** *Cyclophosphamide*, *pyruvate*, *Sperm*, *Mice*

## Abstract

**Background:**

One of the affecting factors in disturbance process of spermatogenesis is chemotherapeutic-induced oxidative stress resulted from cyclophosphamide (CP) treatment which leads to diminished sperm quality via interference in spermatogenesis process.

**Objective:**

This study was conducted to investigate the effects of ethyl pyruvate (EP) in reducing the CP-induced side effects on reproductive system.

**Materials and Methods:**

24 mature male mice were randomly divided into 3 equal groups and were undergone therapy for 35 days. Control group received normal saline (0.1 ml/day, IP). CP group were injected CP (15 mg/kg/week, IP) and CP+EP group received EP (40 mg/kg/day, IP) as well as CP. In the end of the treatment period, the mice were euthanized by cervical dislocation. Then, the epididymis was incubated with CO_2_ in a human tubal fluid medium (1 ml) for half an hour in order to float sperm. Then, the number, motility, viability (eosin-nigrosin staining), DNA breakage (acridine orange staining), nucleus maturity, and sperm morphology (aniline blue staining) were analyzed.

**Results:**

The average (15.87±1.28), motility (35.77±2.75), viability (40±3.03), nucleus maturity (36±2.79) and sperm morphology (61.75±0.85) were decreased significantly in CP group in comparison with control and EP groups, whereas EP caused significant increase of these parameters. Also, the percentage of DNA damage was increased significantly in CP group (41.75±3.75) in comparison with control (2±0.71) and EP groups (22.5±4.13).

**Conclusion:**

The results of this study revealed ameliorating effects of EP on sperm quality of CP treated animals.

## Introduction

Chemotherapy is a common medication for preventing the development as well as suppressing tumor cells. In this therapeutic process the bodies other fast-growing cells are destroyed in addition to cancer cells (1). Despite enhancing functions of chemotherapy, studies have shown that these cytotoxic factors lead to the production of free radicals which cause side effects of these drugs (2). Chemicals with deleterious effects on spermatogenesis lead to production of damaged gem cells from standpoint of quality and function. These cells in turn, lead to congenital anomalies, fetal mortality or background to develop cancer (3).

Cyclophosphamide (CP) is one of the most common drugs used in chemotherapy which is produced and used to treatment of tumors as well as its beneficial effects in controlling tumors and cancer tissues, it has many side effects. Reproductive system poisoning is the major side effect of CP in humans and animals (4, 5). The metabolism of CP is completed by microsomal enzymes of the liver, and CP is transformed to its active metabolites phosphor amide mustard and acrolein (6). Acrolein is a toxic metabolite of CP that disturbs antioxidant system of tissues and produces high levels of reactive oxygen species (ROS) (7, 8).

The status in which the levels of oxidants are high and/or the levels of antioxidants are low in cells is called oxidative stress. In this condition the concentration of oxygen free radicals is higher than their biological amounts (9, 10). Thus, CP-produced free radicals in the cells cause oxidative stress in the body especially in reproductive system and newly forming and maturing spermatozoa in testis and epididymis leads to decrease of fertility in men undergoing the treatment (5, 11, 12). On the other hand, spermatozoa due to high contents of polyunsaturated fatty acids as well as low antioxidant capacity are more sensitive to oxidative damages (13). Meanwhile, the investigations have shown that oxidative damages induced by CP arise from hydrogen peroxide production (14). In the same line, other papers have reported the role of antioxidants in reducing DNA damage and apoptosis in spermatozoa as well as increasing fertility (15, 16).

A study revealed that Satureja Khuzestanica extract because of its antioxidant properties could ameliorate sperm quality in the CP treated mice (17). Another study also showed that Achillea Millefolium extract prevents toxic side effects of the CP on sperm quality parameters in rats (18). Bearing in mind that antioxidative compounds can protect cells against harmful free radicals produced in the chemotherapy process in this study we used Ethyl pyruvate (EP) as a synthetic antioxidant. Because of its instability there is a limitation in using pyruvate as a drug. So, we used EP consisted of pyruvic acid and ethanol. Pyruvate works in the cell through removing ROS (19-21).

In addition, EP has several therapeutic applications including; as a suppressor of tumor development a therapeutic factor in acute pulmonary diseases and an ameliorating agent of disorders associated with hemorrhagic shock in the body (22-24). Ethyl pyruvate caused improvement in sperm quality on the Methotrexate-treated mice (25). Because, the effect of EP on the sperm quality in the CP treated mice has not been studied so far, this study was carried out to evaluate the antioxidative effects of EP on parameters of semen including the number, motility, viability, DNA damage level, and nucleus maturity of spermatozoa.

## Materials and methods

**Groups and treatment**

In this exprimental study that has been done in Veterinary faculty of Urmia University in 2013, 24 mice were randomly divided in to three groups. The control group received normal saline (0.2 ml, IP). The CP group received CP (15 mg/kg/week, IP) (26). CP+EP group were administrated with CP as well as EP (40 mg/kg/day, IP) (27).

**Chemicals**

Cyclophosphamide was supplied by human drug store (Baxter, Germany 500 mg) and EP was purchased from SIGMA-USA-ALDRICH-E47808.

**Animals and treatment groups**

In this experimental study, 24 male healthy mice (NMRI) aged 8-12 weeks were used. These mice were kept in standard conditions including temperature 22±2^o^C, humidity 30-60%, and light/dark cycle of 14 and 10 hours respectively. The experimental project was approved by ethics committee of Urmia University, Urmia, Iran. During the course of this experiment, we followed the recommendations by our Institutional Animal Care and Use Committee for the handling, maintenance, treatment, and killing of the animals.

**Sperm sampling**

Following the treatment course (35 days), the animals were euthanized with ketamine over dose (100 mg kg^-1^).Then, both epididymis (cauda and vas) of each mouse were transferred to a 60 mm Petri dish containing 1 ml Human Tubal Fluid (HTF; Sigma, St. Louis, USA) culture and 4 mg mL^-1^ bovine serum albumin (BSA; Sigma, St. Louis, USA) medium pre-warmed to 37^o^C. The caudate was minced making 5-7 slashes with a 30-gauge needle of an insulin syringe. After 30 min of incubation at 37^o^C in 5% CO_2_, spermatozoa were released from epididymis (28).

**Assessment of sperm count**

After preparing a 1:20 dilution of spermatozoa using distilled water, 10µl of the solution was transferred on to a Neobar slide which a stone cover slip had been placed on it before and then spermatozoa were counted (29).

**Evaluation of spermatozoa motility**

A volume of 10 µl of sperm containing culture medium was placed on a Neobar slide and was covered with as tone cover glass. Sperm motility was assessed by using a light microscope at 10× and 20× magnifications (30).

**Evaluation of sperm viability**

Twenty microliters of the sperm sample was placed on a clean slide and then 20 µl eosin solution was added. After 20-30 sec 20 µl nigrosin was further added. After preparing smears and drying slides, the percentages of alive (colorless) and dead (colored with eosin) spermatozoa were determined with a light microscope (at magnification of 10-40×) (29).

**DNA strand damage level evaluation**

DNA damage was observed as yellow to red fluorescence based on the level of damage by using acridine orange staining. In this method, semen was washed three times with PBS and after removing supernatant liquid, obtained sediment was reached final volume using PBS. Then, smear was prepared from sperm culture and after drying in lab, was sunken in acetone/ ethanol (1:1) solution. After air drying the slides were immersed in acridine orange for 7 min. After final drying in a dark place the slides were assessed using a fluorescence microscope with a 100× lens and the results were reported as percentage (31).

**Sperm chromatin condensation evaluation**

Similar to the above method, after stabilizing in ethanol-acetone solution and drying in the air, the slides were placed for 7 min in aniline blue solution and again after drying were examined by alight microscope with 10×100 magnification. Thus, immature spermatozoa appeared grayish dark blue due to high histone levels but mature spermatozoa were stained pale (31).

**Morphological evaluation of spermatozoa**

Two staining methods were used, including aniline blue and eosin-nigrosin. Aniline blue staining was used for counting abnormal appearance spermatozoa, while for counting spermatozoa containing cytoplasmic residues (immature spermatozoa) the eosin-nigrosin staining was applied (29).

**Statistical analysis**

The data were analyzed by SPSS (Version 20; SPSS Inc., Chicago, Illinois, USA) and one-way ANOVA and Tukey test for comparing the pair groups independently were used. P<0.05 was considered significant.

## Results

The average number of spermatozoa in CP group in comparison with control and CP+EP groups revealed a decrease. In this study it was shown that EP could increase the average number of spermatozoa significantly comparing to the CP group (Table I). The percentage of motile spermatozoa in the groups indicates that there was a significant decrease in the CP group in comparison with the control group and CP+EP groups. In this case also EP caused a significant increase in sperm motility in the CP+EP group comparing to the CP group (Table I).

Following the staining with aniline blue the number of immature spermatozoa was calculated and results suggested a significant increase in the percentage of immature spermatozoa in the CP group relative to control and CP+EP groups. The CP+EP group showed a significant decrease in comparison with the CP group (Table II). Current study reveals that the fraction of damaged, single stranded and broken DNA in the CP group had a significant increase relative to the control group, and in the CP+EP group it was significantly decreased in comparison with the CP group (Table II). In this investigation the spermatozoa with normal morphology suggested a significant drop in the CP group compared to the control group and the CP+EP group.In this case also the CP+EP group showed a significant reduction of malformed spermatozoa in comparison with the CP group. The percentage of live spermatozoa that were stained with eosin- nigrosin method indicated a significant decrease in the CP group relative to the control and EP groups. EP could increase this parameter significantly in the CP+EP group compared to the other groups (Table I).

**Table I:** Different parameters of sperm quality in different groups

**Groups**	**Sperm count ** **(×10** ^6 ^ **ml) NO**	**Sperm motility** %	**Sperm viability** %	**Normal sperms** %
Control	32 ± 0.65	61 ± 2.12	68.5 ± 0.64	92.25 ± 0.85
CP	15.87 ± 1.28^a(p<0.001)^	35.77 ± 2.75^a(p<0.001)^	40 ± 3.03 ^a(p=0.03)^	61.75 ± 0.85^a(p<0.001)^
CP + EP	20.75 ± 1.36^a(p<0.001)^	49.50 ± 2.02^ab(p<0.001, p=0.08)^	53 ± 4.6 ^ab(p<0.001, p=0.01)^	77.25 ± 2.14^ab(p<0.001, p<0.001)^

CP: Cyclophosphamide, EP: Ethyl pyruvate

Data are presented as mean±SE.

The letters of “a” and “b” in front of data indicate significantly differences with control and CP groups respectively ( Student’s tukey test ).

**Table II:** Mean percentage of sperm chromatin condensation and DNA disintegrity in different groups

**Groups**	**Chromatin condensation (AB+)**	**DNA integrity of sperms (AO+)**
Control	2±0.71	2±0.41
CP	41.75±3.75 ^a (p<0.001)^	36±2.79 ^a (p<0.001)^
CP+ EP	16.5±1.04 ^ab (p<0.001, p=0.01)^	22.5±4.13 ^ab (p<0.001, p<0.001)^

CP: Cyclophosphamide, EP: Ethyl pyruvate

Data are presented as mean±SE.

AB: Aniline blue

AO: Acridine orange.

The letters of “a” and “b” in front of data indicate significantly differences with control and CP groups respectively ( Student’s tukey test ).

**Figure 1:**
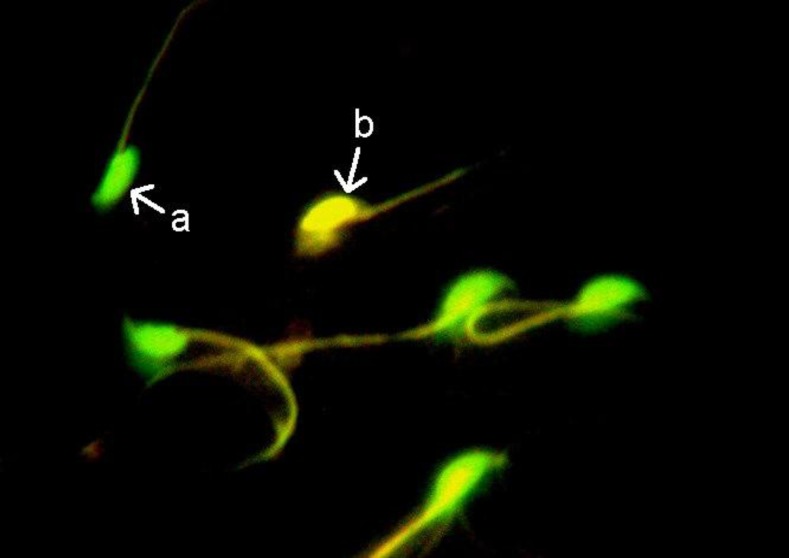
Acridine orange (AO) staining for studying of DNA damaged sperm; a, green sperm head and b, yellow sperm head shows healthy and damaged sperm, respectively (×100 eyepiece magnification).

**Figure 2:**
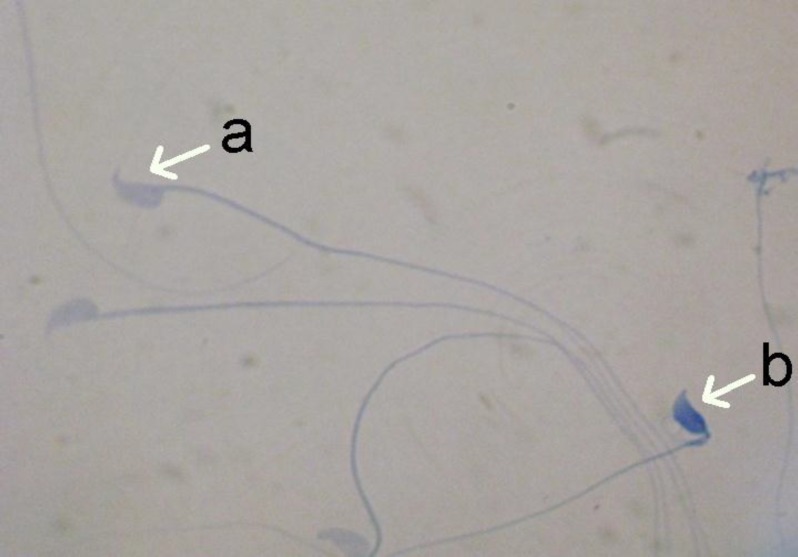
Aniline blue (AB) staining; pale blue and dark blue indicates mature and immature sperms, respectively (×100 eyepiece magnification).

## Discussion

Despite the beneficial therapeutic effects of chemotherapy, it causes severe oxidative stress. Current study looks for a way to damp the toxic side effects of the drugs. This study showed that the mean number of sperms was increased significantly in the EP+CP group compared to the CP group (p<0.05). Crocin (extract of saffron) with its antioxidant properties could increase the number of spermatozoa in the CP-treated mice (32). Toxic side effects of CP on spermatocytogenesis and spermiogenesis and so on germinal epithelium destruction could be the result of decrease of sperm generation (33).

Sperm motility reduction in the CP group compared to the other groups in the present study is probably related to generation of ROS by CP since the produced ROS in mitochondria caused mitochondrial DNA damage on middle piece of spermatozoa all of these deleterious effects may be able to decrease sperm motility (14, 34, 35). This study showed that the mean percentage of DNA damaged and DNA broken sperm in CP group has been increased significantly (p<0.05), probably this process was resulted from oxidative stress induced by ROS generation (36-38). Some studies confirmed the beneficial effects of antioxidants in decreasing of sperm DNA damage then the significant increase of this parameter in the EP+CP group compared to the CP group was probably related to antioxidant properties of EP (15, 16). Also observation of significant increase in mean number of normal morphology and viability in the EP+CP group compared to the CP group could be caused by decreasing of ROS by EP, because according to previous studies, the rate of ROS generation is directly related to the number of dead or abnormal spermatozoa (39). Study of sperm nucleus maturation showed that this parameter increased significantly in the EP+CP group in comparison with the CP group. One of the protective factors of sperm DNA against the oxidative stress is condensation of DNA (protamination) and conversely the increasing of ROS causes changes in all bases, deletion and uncoupling of complement bases, morphological and cross junction changes of DNA and changes in reorganization of chromosomes (40, 41). Then, decreasing of mean percentage of spermatozoa with immature nucleus is a robust reason for decreasing the rate of ROS by EP antioxidant effects.

All previous studies that have been done on CP state that this drug has some deleterious effects on reproductive system and diminishes sperm quality. These results are in-line with the results of the current study. Meanwhile, EP was able to modulate all parameters of ROS-affected sperm including count, motility, viability, DNA breakage, nucleus maturity, and sperm morphology. Of course, this enhancement was not in the level of the control group. With regard to beneficial applications of ROS production, this study investigated the anti-oxidative role of EP to regulate CP side effects on the reproductive system and sperm quality (19-21).

Due to its instability, application of pyruvate is limited. So, EP which consists of pyruvic acid and ethanol is used. EP is a major anti-inflammatory and anti-oxidative stress agent that has therapeutic characteristics in different conditions including protective effects on nerves against parquet poisoning a powerful supporter against spinal cord ischemic injury through inhibiting secretion of HMGB_1_ (42-44).

## Conclusion

Production of high amounts of free radicals in chemotherapies including CP with inducing oxidative stress in the body, leads to the poisoning of the reproduction system and reducing sperm quality in patients under treatment. This study reveals that using EP as an antioxidant leads to an increase in sperm quality in CP-treated animals through creating a protection against the undesirable effects of the CP.

## Note

***This article extracted from M.Sc. thesis. (Zahra Bakhtiary)***
